# Antihypertensive Treatment Differentially Affects Vascular Sphingolipid Biology in Spontaneously Hypertensive Rats

**DOI:** 10.1371/journal.pone.0029222

**Published:** 2011-12-15

**Authors:** Léon J. A. Spijkers, Ben J. A. Janssen, Jelly Nelissen, Merlijn J. P. M. T. Meens, Dayanjan Wijesinghe, Charles E. Chalfant, Jo G. R. De Mey, Astrid E. Alewijnse, Stephan L. M. Peters

**Affiliations:** 1 Department of Pharmacology & Pharmacotherapy, Academic Medical Center, Amsterdam, The Netherlands; 2 Department of Pharmacology, Maastricht University, Maastricht, The Netherlands; 3 Department of Biochemistry, Virginia Commonwealth University, Richmond, Virginia, United States of America; City of Hope National Medical Center and Beckman Research Institute, United States of America

## Abstract

**Background:**

We have previously shown that essential hypertension in humans and spontaneously hypertensive rats (SHR), is associated with increased levels of ceramide and marked alterations in sphingolipid biology. Pharmacological elevation of ceramide in isolated carotid arteries of SHR leads to vasoconstriction via a calcium-independent phospholipase A_2_, cyclooxygenase-1 and thromboxane synthase-dependent release of thromboxane A_2_. This phenomenon is almost absent in vessels from normotensive Wistar Kyoto (WKY) rats. Here we investigated whether lowering of blood pressure can reverse elevated ceramide levels and reduce ceramide-mediated contractions in SHR.

**Methods and Findings:**

For this purpose SHR were treated for 4 weeks with the angiotensin II type 1 receptor antagonist losartan or the vasodilator hydralazine. Both drugs decreased blood pressure equally (SBP untreated SHR: 191±7 mmHg, losartan: 125±5 mmHg and hydralazine: 113±14 mmHg). The blood pressure lowering was associated with a 20–25% reduction in vascular ceramide levels and improved endothelial function of isolated carotid arteries in both groups. Interestingly, losartan, but not hydralazine treatment, markedly reduced sphingomyelinase-induced contractions. While both drugs lowered cyclooxygenase-1 expression, only losartan and not hydralazine, reduced the endothelial expression of calcium-independent phospholipase A_2_. The latter finding may explain the effect of losartan treatment on sphingomyelinase-induced vascular contraction.

**Conclusion:**

In summary, this study corroborates the importance of sphingolipid biology in blood pressure control and specifically shows that blood pressure lowering reduces vascular ceramide levels in SHR and that losartan treatment, but not blood pressure lowering per se, reduces ceramide-mediated arterial contractions.

## Introduction

Sphingolipids are a class of bioactive lipids with important roles in cell signaling as they control cell growth, migration and differentiation [1]–[4]. It is becoming increasingly evident that these lipids play an important physiological role in the cardiovascular system. In the vasculature for instance, the sphingolipid sphingosine-1-phosphate (S1P) is known to regulate endothelial function via activation of nitric oxide synthase [5], [6] or inhibition of endothelium-derived hyperpolarizing factors [7].

More recently it was shown that sphingolipids also play a pathological role in hypertension. For instance, a recent genetic analysis by Fenger *et al.* suggested the involvement of the sphingolipid system in the regulation of blood pressure and hypertension on a genetic basis [8]. Moreover, Yogi *et al.* have recently shown *in vitro* that S1P is a potent inducer of pro-inflammatory signaling pathways through epidermal growth factor receptor and platelet-derived growth factor trans-activation, a pathway that is up-regulated in spontaneously hypertensive stroke-prone rats [9]. We have previously shown that sphingolipids are involved in the pathophysiology of hypertension *in vivo* [10]. This latter study showed that vascular and plasma levels of the bioactive sphingolipid ceramide were significantly higher in spontaneously hypertensive rats (SHR) than in normotensive Wistar Kyoto (WKY) rats. In addition, we demonstrated that also in humans with essential hypertension, plasma ceramide levels correlated positively to the level of blood pressure. The pathophysiological relevance of this finding was demonstrated by the observation that pharmacological elevation of ceramide in isolated carotid arteries of SHR but not WKY, leads to endothelium-dependent arterial contractions by thromboxane A_2_ (TXA_2_) and increases blood pressure in SHR *in vivo* [10]. Thus sphingolipids are not only involved in the regulation endothelium-derived relaxing factors but also the production of endothelium-derived contracting factors, especially in the setting of hypertension, and may thus contribute to endothelial dysfunction.

Aforementioned data clearly indicate that hypertension is associated with marked alterations in vascular sphingolipid biology. In the present study we investigated whether these alterations in sphingolipid biology could be reversed by antihypertensive treatment. We investigated the effects of chronic (4-week) treatment with the angiotensin II type 1 antagonist losartan or the non-selective vasodilator hydralazine in SHR on ceramide levels in vascular tissue and blood plasma. Furthermore, we investigated the effects of both antihypertensive treatment regimens on ceramide-mediated, endothelium-dependent arterial contractions induced by exogenously applied sphingomyelinase (SMase). In this study, we observed that both antihypertensive drugs significantly lowered arterial ceramide levels in concurrence with the reduced blood pressure, and that losartan, but not hydralazine, inhibited SMase-induced contractions of isolated carotid arteries, most likely by decreasing the expression of endothelial calcium-independent PLA_2_ (iPLA_2_).

## Methods

### Ethics statement

The animal experiments performed in this study followed a protocol that was specifically approved by the Animal Ethics Committee of Maastricht University, Maastricht, The Netherlands (approval number: 2010-050), and was in accordance with EU guidelines (2010/63/EU) on the care and use of laboratory animals.

### Compounds and antibodies

Acetyl-β-methylcholine (methacholine; MCh) and phenylephrine (Phe) were purchased from Sigma-Aldrich Chemical Co. (St. Louis, MO, USA) and neutral sphingomyelinase C (SMase; from *Staphylococcus aureus*) from Biomol International L.P. (Plymouth, PA, USA). Antibodies against cyclooxygenase-1 (order#160109; 1/400 dilution used) and thromboxane synthase (#160715; 1/200) were purchased from Cayman Chemical; calcium-independent phospholipase A_2_ antibody (#ab23706; 1/400) from Abcam (Cambridge, UK) and Von Willebrand factor antibody (GTX74830; 1/200) from GeneTex (Irvine, CA, USA). Alexa Fluor 488-labeled (#A-11029: 1/400) and Alexa Fluor 546-labeled secondary antibodies (#A-11010: 1/400) were from Invitrogen (Carlsbad, CA, USA).

### Animals and treatment

Adult six month old male spontaneously hypertensive rats (SHR) were purchased from Charles River (L'Arbresle, France). Rats were anesthetized with isoflurane and osmotic minipumps (2ML4; Alzet, California, USA) were subcutaneously implanted. The minipumps were filled with losartan (dissolved in saline), or hydralazine (dissolved in saline). The concentrations were chosen to obtain the continuous 4 weeks release of losartan at 20 mg/kg.day and hydralazine at 9 mg/kg.day. Because the maximal solubility of hydralazine was reached in saline, additional hydralazine was added to the drinking water to increase the dose to 20 mg/kg.day. In untreated SHR a dummy device (PE tube of the same size as the 2ML4 pumps) was implanted subcutaneously.

### Blood pressure measurements

Conscious tail cuff blood pressure measurements were performed 28 days after the initiation of the drug treatments using the CODA^tm^ system (Kent Scientific Corporation, CT, USA). Differences in tail cuff systolic blood pressures (SBP) were verified by intra-arterial measurements when rats were anesthetized with 2.5% isoflurane. For this purpose a PE-10 canula was inserted into the abdominal aorta via the femoral artery. The arterial pressure was recorded at 2.5 kHz using IDEEQ data acquisition software (Maastricht, The Netherlands). When blood pressure was stabilized, baseline values of blood pressure were recorded and averaged over 10–15 minutes. Hereafter, blood plasma, organs and blood vessels were collected and processed.

### Liquid chromatography - mass spectrometry on blood plasma

Post-anesthesia, the thoracic region was opened and 5 mL of blood was collected by abdominal aorta puncture using a 21G needle (BD Microlance 3) and collected in a pre-chilled (0°C) polypropylene blood collection tube containing PECT solution as described in Spijkers *et al.* [10]. Blood plasma was prepared by centrifugation for 20 min at 1600× g, 4°C within 10 min after collection and stored at −80°C. Furthermore, the thoracic aorta was isolated and snap-frozen in liquid nitrogen. For blood plasma samples, lipids were extracted from 33 µL blood plasma as described by and Merrill *et al.* [11] Wijesinghe *et al.* [12] with slight modifications. Briefly; to 33 µL of plasma 167 µL water, 1 mL methanol and 0.5 mL chloroform were added together with an internal standard containing 500 pmol of the following; d17∶1 sphingosine, sphinganine, sphingosine-1-phosphate and sphinganine-1-phosphate, and d18∶1/12∶0 ceramide, ceramide-1-phosphate, sphingomyelin and glucosylceramide. The mixture was sonicated and incubated at 48°C overnight. The following day, extracts were subjected to base hydrolysis for 2 h at 37°C using 150 µL of 1 M methanolic potassium hydroxyde. Following base hydrolysis the extract was completely neutralized by the addition of 6 µL glacial acetic acid. The neutralization was confirmed by pH measurement. Half of the extract was dried down and resuspended in reversed phase sample buffer (60%A∶40%B) (A = methanol∶water 60∶40 with 5 mM ammonium formate and 1% formic acid, B = methanol with 5 mM ammonium formate and 1% formic acid). To the remainder of the extract 1 mL chloroform and 2 mL water were added, and the lower phase was transferred to another tube, dried down and brought up in normal phase sample buffer (98%A∶2%B). Sphingosine, sphinganine, sphingosine-1-phosphate sphinganine-1-phosphate and ceramide-1-phosphate were quantified via reversed phase HPLC ESI-MS/MS using a Discovery C18 column attached to a Shimadzu HPLC (20AD series) and subjected to mass spectrometric analysis using a 4000 Q-Trap (Applied Biosystems) as described by Wijesinghe *et al.* [12]. Ceramides, sphingomyelins and monohexosyl ceramides were quantified via normal phase HPLC ESI-MS/MS using an amino column (Sigma) as described by Merrill *et al.* [11]. For aorta samples, lipids were extracted from 500 µL of a 10% homogenate of the tissue in PBS according to Merrill *et al.* [11] and Wijesinghe *et al.* [12] with slight modifications. Briefly to 500 µL of the 10% tissue homogenate 2 mL of methanol and 1 mL of chloroform was added together with an internal standard and processed as described above. The inter-day variability is less than 5% while the intraday variability is less than 7%. The accuracy has been previously verified [12].

### Arterial preparation and isometric force recording

Carotid artery segments were isolated from the rats and mounted into a wire myograph for isometric tension measurements as described by Mulders *et al.* [7]. In brief, vessels were allowed to equilibrate and organ bath buffers were replaced every 15 min with carbogen aerated (95% O_2_, 5% CO_2_) Krebs-Henseleit buffer (pH 7.4; in mM: 118.5 NaCl, 4.7 KCl, 25.0 NaHCO_3_, 1.2 MgSO_4_, 1.8 CaCl_2_, 1.1 KH_2_PO_4_ and 5.6 glucose). Two high K^+^-containing Krebs buffer contractions were performed (pH 7.4; in mM: 23.2 NaCl, 100 KCl, 25 NaHCO_3_, 1.2 MgSO_4_, 1.8 CaCl_2_, 1.1 KH_2_PO_4_ and 5.6 glucose) with 30 min washout in between. Then, 0.3 µM phenylephrine was applied to gain a stabile contraction of >60% of the K^+^-induced contraction, and 10 µM of methacholine was added to assess endothelial integrity. After 30 min another high K^+^-Krebs buffer contraction was performed. After 30 min wash-out, the enzyme sphingomyelinase (SMase; 0.1 U/mL) was applied to the organ baths to measure alterations in vasomotor tone during 1 hour. In other arteries, concentration-response curves for methacholine were generated in half-log concentration increments during phenylephrine-induced contractions.

### Immunohistochemistry

Immunohistochemical protein staining and subsequent fluorescence intensity quantification in carotid artery segments of SHR were performed as described previously [10]. In brief, carotid artery segments were collected in ice-cold Krebs buffer directly after dissection and cleaning, rapidly submerged in OCT compound (Sakura, TissueTek) and frozen in liquid nitrogen with subsequent storage at −80°C. Frozen sections (5 µm thick) were cut on a Leica CM3050S cryostat and cold-air dried. Slides were fixed in 100% acetone for 1 min, washed shortly in PBS and incubated with blocking buffer (2% BSA/PBS) for 30 min at room temperature. After a short wash (in 0.1% BSA/PBS), slides were incubated at 4°C overnight with the primary antibody (dissolved in 0.1% BSA/PBS). Following a triple wash in 0.1% BSA/PBS for 15 min, the appropriate Alexa Fluor 546-labeled secondary antibody was applied for 1 hour at room temperature. The antibody against von Willebrand Factor (vWF) was applied for 1 hour at room temperature as endothelium marker and finally Alexa Fluor 488-labeled secondary antibody was applied. Vessel slides were embedded in DAPI-containing mounting medium and imaged using a Nikon Eclipse TE2000-U fluorescence microscope (Plan Fluor ELWD 20× objective, Nikon DXM1200F digital camera) with NIS Elements AR 2.30 software. Quantification of fluorescence (fluorescent light units; FLU) was performed using NIS Elements. Using the vWF endothelial marker region, mean fluorescence intensity of the protein of interest was quantified for the endothelium by normalizing total fluorescence against untreated EC area. Then, the tunica media was selected and mean fluorescence intensity was determined for smooth muscle cells. For both determinations, an intensity threshold as low as possible was selected to exclude background fluorescence and restricting the area of interest to mere tissue. All settings and exposure times were equally applied to all tissue slides.

### Statistical data analysis

SBP, heart rate, organ weights, protein quantification, aortic and blood plasma sphingolipid content, and isometric tension measurements in carotid artery segments are presented as means±SEM with ‘n’ being the number of individual rats. Statistical analyses were performed by one-way ANOVA followed by Bonferroni multiple comparisons test (95% confidence interval). Full concentration response curves were analyzed by one-way repeated measures ANOVA. All statistical analyses were performed using Prism (GraphPad Prism Software, San Diego, CA, USA). Values of p<0.05 were considered to be statistically significant.

## Results

### Antihypertensive effects of losartan and hydralazine in SHR

**Figure 1:**
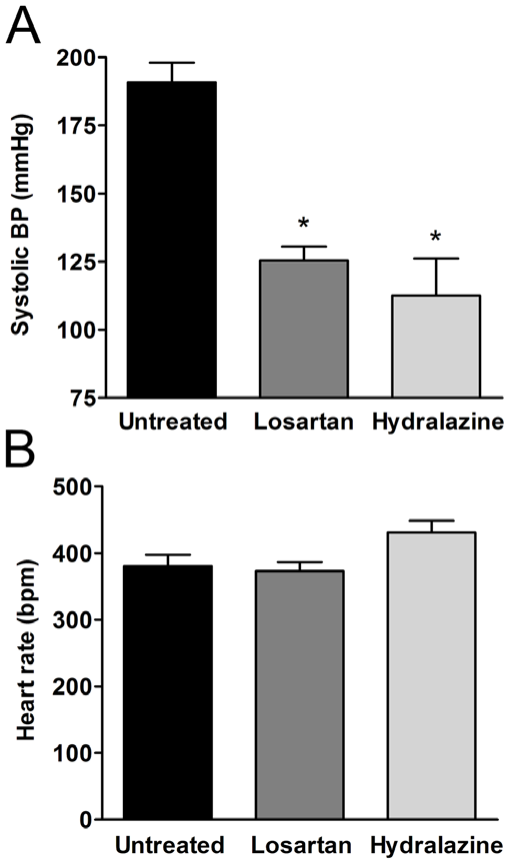
Losartan and hydralazine lower blood pressure in SHR. Hemodynamic parameters of SHR after four weeks untreated or treated with losartan or hydralazine. (**A**) systolic blood pressure; (**B**) corresponding heart rate. Data are expressed as mean±SEM, n = 4–6, * p<0.05 compared to untreated.

The 4-week treatment with either losartan or hydralazine had no significant effect on body weight (Table 1). In untreated conscious SHR, SBP was 191±7 mmHg. The 4 weeks treatment with losartan and hydralazine (at 20 mg/kg.day) reduced tail cuff SBP towards normotensive levels (125±5 mmHg and 113±14 mmHg, respectively) (n = 4–6, P<0.05). Qualitatively similar results were obtained when blood pressures were verified by intra-arterial measurements under isoflurane-anesthetized conditions (128±6, 97±5 and 106±6 mmHg for untreated, losartan and hydralazine treated groups, respectively) (n = 6–8, P<0.05). No significant differences in blood pressure were found between the two treatment groups. Treatment did not significantly change heart rate, although a trend existed for an increased heart rate in the hydralazine group as reported before [13] (Fig. 1B). Treatment with losartan was associated with a significantly greater reduction in normalized heart weight than observed in hydralazine-treated or untreated SHR. No differences in kidney weights were found between the three groups.

**Table 1:** General characteristics of treated and untreated spontaneously hypertensive rats.

	Untreated	Losartan	Hydralazine
Body weight before treatment (g)	335±10	334±7	335±7
Body weight after treatment (g)	356±9	353±7	362±8
Normalized heart weight (mg/g)	4.6±0.2	3.7±0.2*	4.2±0.1
Normalized kidney weight (mg/g)	3.3±0.1	3.2±0.1	3.3±0.1
Carotid artery media/lumen ratio	0.46±0.01	0.39±0.01*	0.38±0.01*

Heart and kidney weight normalized to rat body weight. n = 6–8,

*p<0.05 compared to control.

### Losartan and hydralazine reduce vascular ceramide levels

**Figure 2:**
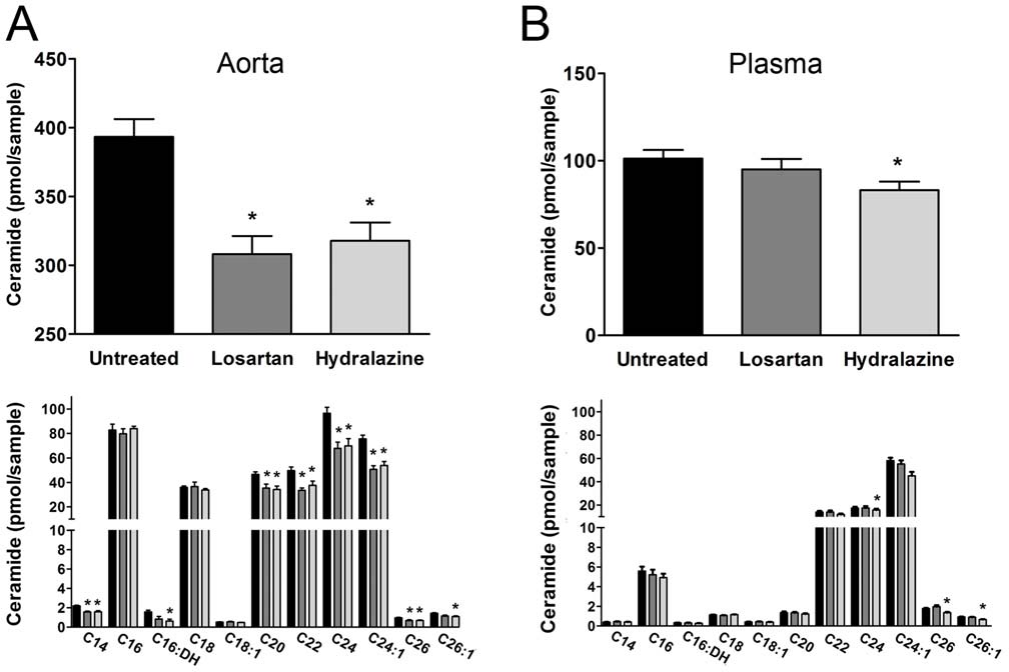
Losartan and hydralazine lower vascular but not plasma ceramide levels in SHR. Ceramide levels (top: total ceramide, bottom: ceramide subspecies) were measured in (**A**) aorta and (**B**) blood plasma of untreated, losartan-treated or hydralazine-treated SHR. Data are presented as mean±SEM, n = 8, * p<0.05. Ceramide subspecies depicted as separated by different tail length. DH: dihydroceramide.

Lipidomic analysis on isolated aortic tissue of SHR indicated that the levels of ceramide were reduced by approximately 20–25%, 4-weeks after treatment with either losartan or hydralazine (Fig. 2A upper panel; n = 8, p<0.05). Most, but not all ceramide subspecies were decreased in vascular tissue after both treatments (Fig. 2A lower panel). This reduction in vascular ceramide levels was not associated with a concomitant reduction of plasma ceramide levels. Only hydralazine reduced (mostly long chain) ceramide levels rather modestly, while losartan had no effect (Fig. 2B).

### Effects of losartan and hydralazine on endothelial function in isolated carotid arteries

**Figure 3:**
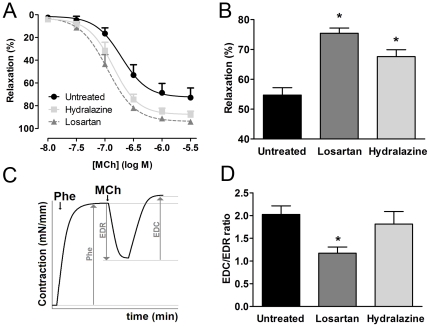
Losartan and hydralazine improve endothelial function in isolated carotid arteries of SHR. (**A**) Concentration-response-curve of methacholine-induced relaxation on phenylephrine pre-constriction. (**B**) Maximal relaxation potential after the addition of a single concentration (10 µM) of methacholine on phenylephrine-induced pre-constriction. (**C**) Schematic representation of a SHR carotid artery response towards a single 10 µM methacholine (MCh) addition on phenylephrine (Phe) precontraction resulting in endothelium-derived relaxation (EDR) and subsequent an additional endothelium-derived contraction (EDC). (**D**) Quantification of the normalized EDC/EDR ratio after 10 µM methacholine addition on phenylephrine pre-contraction as depicted in C. Data are expressed as mean±SEM, n = 7–8, * p<0.05 compared to untreated SHR.

Myograph studies on isolated carotid arteries indicated a significantly improved relaxation in response to methacholine in arteries from animals treated with losartan and hydralazine compared to untreated SHR (Fig. 3A). In phenylephrine pre-contracted carotid arteries of untreated SHR, methacholine (10 µM) induced a maximal relaxation of 55±2% (Fig. 3B). In contrast, in arteries isolated from both losartan-treated and hydralazine-treated SHR, endothelium-dependent relaxations to 10 µM methacholine were significantly enhanced (75±2% and 68±2% respectively, n = 7–8, p<0.05). Although losartan seemed to be somewhat more effective in restoring endothelial function than hydralazine, the difference between the two treatments did not reach statistical significance.

When methacholine is added to the organ bath as a single concentration of 10 µM, in isolated vessels from SHR the endothelium-dependent relaxation is followed by an endothelium-dependent contractile response due to the release of an endothelium-derived contracting factor (EDCF) as depicted schematically in Figure 3C. The ratio between the endothelium-dependent contraction and relaxation provides a more accurate representation of endothelial function. We observed that this ratio was significantly lower in arteries of losartan-treated rats (1.2±0.1) than in arteries of hydralazine-treated (1.8±0.3) or untreated rats (2.0±0.2, n = 7–8, p<0.05) (Fig. 3D).

### Losartan, but not hydralazine treatment, prevents SMase-induced contraction in isolated carotid arteries of SHR

**Figure 4:**
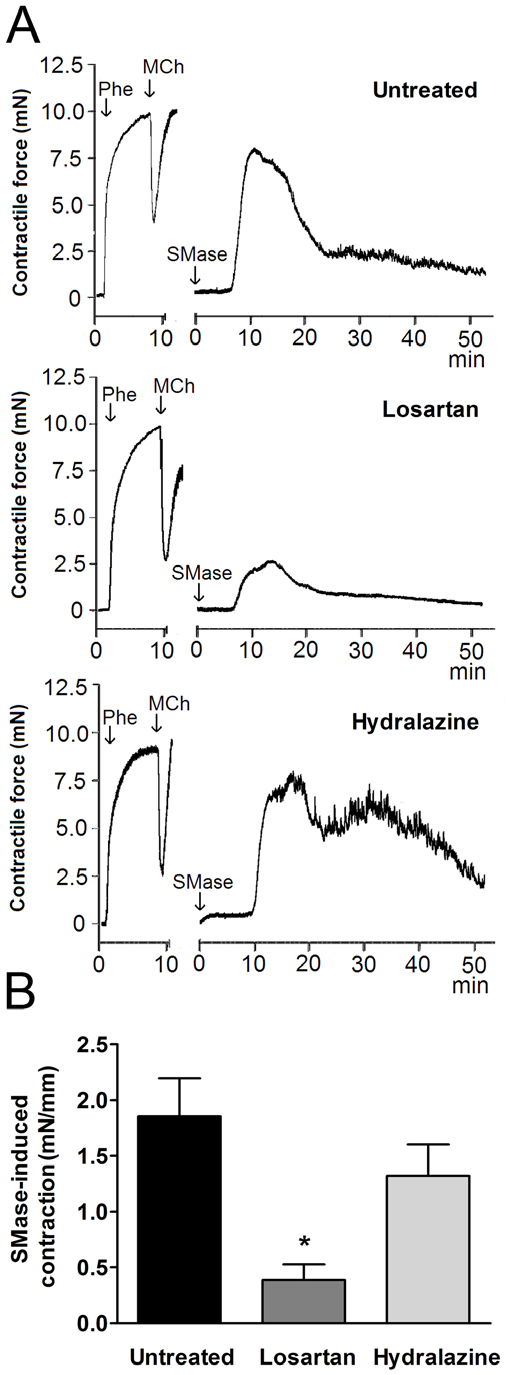
Losartan, but not hydralazine, reduces ceramide-mediated endothelium-dependent contractions in isolated carotid arteries of SHR. (**A**) Typical tracing of 0.1 U/mL sphingomyelinase (SMase)-induced endothelium-dependent contraction in carotid arteries of untreated, losartan-treated and hydralazine-treated SHR. (**B**) Quantification of peak “EDCF-mediated contractile responses” to SMase. Data are expressed as mean±SEM, n = 7–8, * p<0.05 compared to untreated SHR.

Pharmacological elevation of ceramide by exogenous addition of SMase induced a strong contraction of isolated carotid arteries in untreated SHR (1.9±0.3 mN/mm) which was similar to the contractions described previously [10] (Fig. 4). Losartan treatment largely prevented these contractile responses to SMase (0.4±0.1 mN/mm, n = 5, p<0.05). The contractile response to SMase was not altered by hydralazine treatment (1.3±0.3 mN/mm, n = 6, p>0.05) (Fig. 4).

### Losartan, but not hydralazine treatment, reduces endothelial iPLA_2_ expression

**Figure 5:**
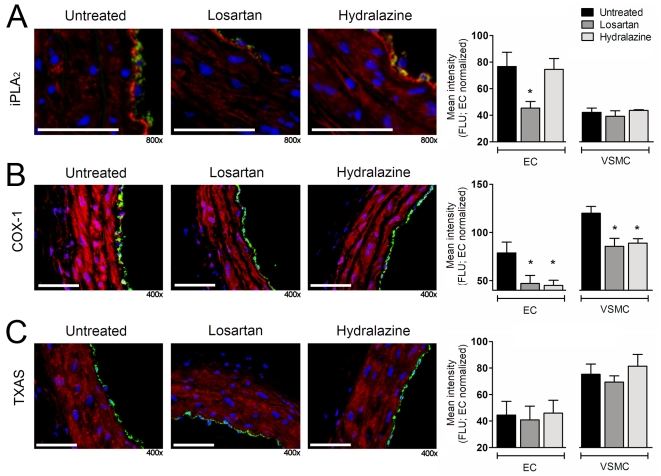
Losartan but not hydralazine reduces endothelial iPLA_2_ expression. (**A**) Immunohistochemical staining (left) and quantification (right) of SHR carotid artery segments depicting cell nuclei staining (blue), with the von Willebrand Factor (vWF) endothelium marker (green) and calcium-independent phospholipase A_2_ (iPLA_2_; red), (**B**) cyclooxygenase-1 (COX-1; red), (**C**) thromboxane synthase (TXAS; red). Scale bars represent 100 µm. Data are expressed as mean±SEM, n = 7–8, * p<0.05 compared to untreated SHR.

We previously described the involvement of calcium-independent phospholipase A_2_ (iPLA_2_), cyclooxygenase-1 (COX-1) and thromboxane synthase (TXAS) in the SMase-induced arterial contractions. These enzymes are up-regulated in the endothelium of carotid arteries from SHR compared to those from normotensive WKY rats [10]. Whereas both losartan as well as hydralazine treatment substantially reduced COX-1 expression in endothelium and smooth muscle cells (Fig. 5B; EC: 47±8 and 45±5 vs 79±11 relative fluorescence (FLU), respectively; VSMC: 86±8 and 89±5 vs 120±7 FLU, respectively; n = 7–8, p<0.05), only losartan reduced endothelial iPLA_2_ expression (Fig. 5A; 45±5 vs 75±8 and 77±11 FLU, respectively; n = 6–7, p<0.05).

The thromboxane A_2_ synthase (TXAS) expression was unaltered after losartan or hydralazine treatment compared to the untreated group in both endothelium (Fig. 5C; 41±10, 46±10 and 45±10 FLU, respectively; n = 7–8, p>0.05) and smooth muscle cell layer (69±5, 81±9 and 75±8 FLU, respectively; n = 7–8, p>0.05).

## Discussion

We have previously shown that hypertension is associated with marked alterations in sphingolipid biology; increased ceramide levels and a predisposition to ceramide-induced endothelium-dependent contractile responses [10]. Here we show that blood pressure lowering by either losartan or hydralazine lowers vascular ceramide levels, and that losartan, but not hydralazine treatment reverses the predisposition to ceramide-induced contractile responses.

The 4-week treatment of SHR with losartan as well as hydralazine, significantly decreased blood pressure as expected, and this was associated with a concomitant lowering of vascular ceramide levels. Since both drugs decreased blood pressure to the same extent but likely via unrelated mechanisms, it can be assumed that the vascular ceramide levels are subject to, and a reflection of, the prevailing blood pressure. The reduction in vascular ceramide levels was not, or only marginally, reflected in plasma ceramide levels. This marginal effect could be the result of detection at an early phase, and a stronger effect may be elicited after prolonged antihypertensive treatment. In addition, it could be that ceramide is released from endothelial cells abluminally, resulting in an increase in basolateral ceramide levels and less systemic spillover towards blood plasma. The exact mechanism of the decreased vascular ceramide levels by blood pressure lowering is currently unknown. Angiotensin signaling has been linked to increases in cellular ceramide levels (for review see Berry *et al.* [14]). However, since these ceramide increasing effects were attributed to AT_2_ receptor stimulation, this does not explain the effects of losartan in the present study. Furthermore, since in addition to losartan also hydralazine was effective in reducing vascular ceramide levels, the reduction in ceramide was, as mentioned before, most likely a consequence of the decreased blood pressure itself. In this regard it is noteworthy that endogenous SMase activity may be potentiated by high shear stress to initiate ceramide production [15]. This may elevate ceramide in hypertension (which on itself may further increase vascular tone in hypertensive subjects) and therefore lower ceramide levels are detected after blood pressure lowering. Although we have measured ceramide levels and investigated sphingolipid biology in larger vessels (i.e., aorta and carotid arteries), we know from our previous *in vivo* experiments [10] that these changes also affect other vascular (resistance) beds. The beneficial effect of losartan and hydralazine on endothelial function of SHR isolated carotid arteries is reflected by the improved potency and efficacy of methacholine-induced relaxation. This finding is in accordance with previous studies showing improvements of endothelial function by treatment with either ACE inhibitors or AT_1_ blockers in several vascular beds [16], [17].

Interestingly, losartan markedly decreased SMase-induced vascular contractions, from which we know that these are endothelium-dependent and most likely mediated by TXA_2_ since these can be inhibited by thromboxane synthase inhibition and TP-receptor antagonism [10]. Hydralazine was ineffective in this respect, suggesting that not blood pressure lowering per se is responsible for this effect. We have previously established that SMase-induced ceramide elevation in the carotid artery of SHR leads to vasoconstriction via an iPLA_2_, cyclooxygenase-1 and thromboxane synthase-dependent mechanism. Thus, the present observation that losartan, but not hydralazine, decreased SMase-mediated endothelium-dependent vascular contractions may be explained by differential effects of these drugs on the expression of the aforementioned proteins in the thromboxane synthesis pathway. Immunohistochemical analysis indicated that losartan as well as hydralazine treatment decreases cyclooxygenase-1 expression in the carotid artery. iPLA_2_ expression, however, was only lowered by losartan treatment. The latter finding may explain the reduced SMase-induced vascular contraction after losartan treatment.

One possible explanation why losartan selectively reduces vascular iPLA_2_ expression could be that protein kinase C (PKC) increases both the activity [18] and the expression [19] of iPLA_2_. Since the angiotensin II type 1 receptor is a potent activator of PKC, and angiotensin II can activate iPLA_2_ in a variety of cell types [20], it is conceivable that angiotensin II increases iPLA_2_ expression in the vasculature. For that reason one would expect that at least angiotensin II receptor antagonists and angiotensin converting enzyme inhibitors would reduce iPLA_2_ expression. Considering the clinical importance of AT_1_ receptor blockade, this new aspect associated with losartan treatment warrants further investigation.

Interestingly, the AT_1_ receptor antagonists losartan and irbesartan, have been shown to possess TP-receptor antagonistic properties [21], [22]. Although this phenomenon may partly explain the beneficial effects of losartan described in the *in vivo* part of this study, it is very unlikely that this interferes with SMase-induced contractions in the isolated carotid arteries. This is because the residual losartan concentrations will be very low once the artery segments are mounted into the organ baths because the drug easily diffuses out of the isolated arteries during myography experiments that last several hours. This has been proven experimentally in a study of Matsumoto *et al.* [23], where losartan-treated rats showed no residual antagonistic effect in isolated arteries on the TP receptor, as indicated by unaffected U46619 concentration-response curves compared to controls in comparable myography experiments.

This study demonstrates a clear link between hypertension and sphingolipid biology and as such it represents a new pathophysiological mechanism in endothelial dysfunction and blood pressure regulation. This pathophysiological mechanism might also be of relevance for new drugs entering the market that target the sphingolipid system (such as the recently approved immunosuppressant FTY720 [24]) or drugs that modulate sphingolipid metabolism and increase ceramide levels as a side effect (such as VEGF antagonists [25]). Indeed, these drugs are known to increase blood pressure in both experimental and clinical settings.

This study also demonstrates that losartan has some unique properties that prevent ceramide-mediated endothelium-dependent vasoconstriction in arteries from SHR and thus may improve endothelial function, which receives a growing interest in hypertension treatment [26]–[29], via this alternative pathway. This phenomenon gives rise to some interesting new questions such as whether this is a unique property of losartan or that other AT_1_ blockers, RAAS inhibitors or unrelated antihypertensive drugs share the same properties. Future studies are warranted to answer these questions and whether the effect of losartan, and possibly other drugs, on reducing iPLA_2_ expression are a beneficial contribution to antihypertensive treatment options, especially in conjunction with disease states that have been reported to be associated with increased ceramide levels and signaling such as diabetes and obesity [30], [31].

In conclusion, this study corroborates the association between blood pressure and alterations in sphingolipid biology by showing that vascular ceramide levels are sensitive to antihypertensive therapy. In addition, it demonstrates that losartan can improve endothelial function via inhibition of ceramide-mediated endothelium-dependent vasoconstriction.

## References

[1] Chatterjee S (1998). Sphingolipids in atherosclerosis and vascular biology.. Arterioscler Thromb Vasc Biol.

[2] Gulbins E (2003). Regulation of death receptor signaling and apoptosis by ceramide.. Pharmacol Res.

[3] Pyne S, Pyne NJ (2000). Sphingosine 1-phosphate signalling in mammalian cells.. Biochem J.

[4] Saba JD, Hla T (2004). Point-counterpoint of sphingosine 1-phosphate metabolism.. Circ Res.

[5] Mulders AC, Hendriks-Balk MC, Mathy MJ, Michel MC, Alewijnse AE (2006). Sphingosine kinase-dependent activation of endothelial nitric oxide synthase by angiotensin II.. Arterioscler Thromb Vasc Biol.

[6] Dantas AP, Igarashi J, Michel T (2003). Sphingosine 1-phosphate and control of vascular tone.. Am J Physiol Heart Circ Physiol.

[7] Mulders AC, Mathy MJ, Meyer zu Heringdorf D, ter Braak M, Hajji N (2009). Activation of sphingosine kinase by muscarinic receptors enhances NO-mediated and attenuates EDHF-mediated vasorelaxation.. Basic Res Cardiol.

[8] Fenger M, Linneberg A, Jorgensen T, Madsbad S, Sobye K (2011). Genetics of the ceramide/sphingosine-1-phosphate rheostat in blood pressure regulation and hypertension.. BMC Genet.

[9] Yogi A, Callera GE, Aranha AB, Antunes TT, Graham D (2011). Sphingosine-1-phosphate-induced inflammation involves receptor tyrosine kinase transactivation in vascular cells: upregulation in hypertension.. Hypertension.

[10] Spijkers LJ, van den Akker RF, Janssen BJ, Debets JJ, De Mey JG (2011). Hypertension is associated with marked alterations in sphingolipid biology: a potential role for ceramide.. PLoS ONE.

[11] Merrill AH, Sullards MC, Allegood JC, Kelly S, Wang E (2005). Sphingolipidomics: high-throughput, structure-specific, and quantitative analysis of sphingolipids by liquid chromatography tandem mass spectrometry.. Methods.

[12] Wijesinghe DS, Allegood JC, Gentile LB, Fox TE, Kester M (2010). Use of high performance liquid chromatography-electrospray ionization-tandem mass spectrometry for the analysis of ceramide-1-phosphate levels.. J Lipid Res.

[13] Demirci B, McKeown PP, Bayraktutan U (2005). Blockade of angiotensin II provides additional benefits in hypertension- and ageing-related cardiac and vascular dysfunctions beyond its blood pressure-lowering effects.. J Hypertens.

[14] Berry C, Touyz R, Dominiczak AF, Webb RC, Johns DG (2001). Angiotensin receptors: signaling, vascular pathophysiology, and interactions with ceramide.. Am J Physiol Heart Circ Physiol.

[15] Czarny M, Schnitzer JE (2004). Neutral sphingomyelinase inhibitor scyphostatin prevents and ceramide mimics mechanotransduction in vascular endothelium.. Am J Physiol Heart Circ Physiol.

[16] Dohi Y, Criscione L, Pfeiffer K, Luscher TF (1994). Angiotensin blockade or calcium antagonists improve endothelial dysfunction in hypertension: studies in perfused mesenteric resistance arteries.. J Cardiovasc Pharmacol.

[17] Tschudi MR, Criscione L, Novosel D, Pfeiffer K, Luscher TF (1994). Antihypertensive therapy augments endothelium-dependent relaxations in coronary arteries of spontaneously hypertensive rats.. Circulation.

[18] Akiba S, Mizunaga S, Kume K, Hayama M, Sato T (1999). Involvement of group VI Ca2+-independent phospholipase A2 in protein kinase C-dependent arachidonic acid liberation in zymosan-stimulated macrophage-like P388D1 cells.. J Biol Chem.

[19] Xie Z, Gong MC, Su W, Xie D, Turk J (2010). Role of calcium-independent phospholipase A2beta in high glucose-induced activation of RhoA, Rho kinase, and CPI-17 in cultured vascular smooth muscle cells and vascular smooth muscle hypercontractility in diabetic animals.. J Biol Chem.

[20] Xie Z, Gong MC, Su W, Turk J, Guo Z (2007). Group VIA phospholipase A2 (iPLA2beta) participates in angiotensin II-induced transcriptional up-regulation of regulator of g-protein signaling-2 in vascular smooth muscle cells.. J Biol Chem.

[21] Li P, Ferrario CM, Brosnihan KB (1998). Losartan inhibits thromboxane A2-induced platelet aggregation and vascular constriction in spontaneously hypertensive rats.. J Cardiovasc Pharmacol.

[22] Aulakh GK, Sodhi RK, Singh M (2007). An update on non-peptide angiotensin receptor antagonists and related RAAS modulators.. Life Sci.

[23] Matsumoto T, Ishida K, Nakayama N, Taguchi K, Kobayashi T (2010). Mechanisms underlying the losartan treatment-induced improvement in the endothelial dysfunction seen in mesenteric arteries from type 2 diabetic rats.. Pharmacol Res.

[24] Brinkmann V (2009). FTY720 (fingolimod) in Multiple Sclerosis: therapeutic effects in the immune and the central nervous system.. Br J Pharmacol.

[25] Petrache I, Natarajan V, Zhen L, Medler TR, Richter AT (2005). Ceramide upregulation causes pulmonary cell apoptosis and emphysema-like disease in mice.. Nat Med.

[26] Hu ZP, Wang BN, Qian HY, Zhou Q, Wei W (2010). Fixed-dose telmisartan/hydrochlorothiazide in comparison with losartan/hydrochlorothiazide in decreasing serum hepatocyte growth factor and improving endothelial dysfunction in hypertensive patients.. Int Heart J.

[27] Modena MG, Bonetti L, Coppi F, Bursi F, Rossi R (2002). Prognostic role of reversible endothelial dysfunction in hypertensive postmenopausal women.. J Am Coll Cardiol.

[28] Ruschitzka F, Noll G, Luscher TF (1999). Angiotensin converting enzyme inhibitors and vascular protection in hypertension.. J Cardiovasc Pharmacol.

[29] Taddei S, Virdis A, Ghiadoni L, Salvetti A (1998). The role of endothelium in human hypertension.. Curr Opin Nephrol Hypertens.

[30] Haus JM, Kashyap SR, Kasumov T, Zhang R, Kelly KR (2009). Plasma ceramides are elevated in obese subjects with type 2 diabetes and correlate with the severity of insulin resistance.. Diabetes.

[31] Boini KM, Zhang C, Xia M, Poklis JL, Li PL (2010). Role of sphingolipid mediator ceramide in obesity and renal injury in mice fed a high-fat diet.. J Pharmacol Exp Ther.

